# Supplementary material for: Antarctic Thraustochytrids as Sources of Carotenoids and High-Value Fatty Acids
Source: Mar Drugs. 2021 Jul 6;19(7):386. doi: 10.3390/md19070386 (PMC8303828; doi:10.3390/md19070386)
Supplement: Supplementary file 1 [file marinedrugs-19-00386-s001.zip › Leyton et al 2021 Supplemetal Material revised - al.pdf]

SUPPLEMENTAL MATERIAL

Antarctic thraustochytrids as sources of carotenoids and high-value fatty acids

Allison Leyton, Liset Flores, Carolina Shene, Yusuf Chisti, Giovanni Larama, Juan A. Asenjo, Roberto E. Armenta

Table S1. Some morphological characteristics of the isolated thraustochytrid strains. Colony texture: soft (S) or firm (F); colony size: punctiform (P) or small (S); color of the colony: white (W), light yellow (LY), or orange (O); growth in liquid medium (LM) as: individual (I) or grouped (G) cells; presence of motile zoospores (MZ).

Table S2. Effects of initial glucose concentration (G_0) on biomass concentration (X) and the content of total lipids (TL) and carotenoids (TC) in biomass of *Thraustochytrium* sp. RT2316-16 after 5 days of culture.

Table S3. *Thraustochytrium* sp. RT2316-16 genes annotated for biosynthesis, elongation and desaturation of fatty acids.

Table S4. *Thraustochytrium* sp. RT2316-16 genes annotated for biosynthesis of terpenoid backbone and carotenoids.

Figure S1. Light microscope image (40×) of some of the isolated thraustochytrid strains. (a) RT2316-29, (b) RT2316-15, (c) RT2316-45 and (d) RT2316-16.

Figure S2. Phylogenetic analysis of Antarctic thraustochytrids isolated from samples collected at different locations in Antarctica during Antarctic Scientific Expedition 54, February 2018 (Tables 1 and 2). Phylogenetic tree was generated by phylogeny.fr [41] (http://www.phylogeny.fr), using MUSCLE, ProtDist/FastDist+BioNJ (distance-based method) and TreeDyn, for multiple sequence alignment, tree construction and tree

visualization, respectively. Names shown in blue are isolates that accumulated carotenoids.

Figure S3. Thin layer chromatogram of carotenoids recovered from *Thraustochytrium* sp. RT2316-16 biomass (lanes 1–3) and authentic standards (astaxanthin, lane a; canthaxanthin, lane b; β-carotene, lane c).

Figure S4. Distribution of enzymes among the metabolic pathways of *Thraustochytrium* sp. RT2316-16, based on the enzyme genes identified in the genome. Results were obtained using KEGG Mapper Reconstruction tool.

Figure S5. Distribution of enzymes within the following metabolisms: carbohydrates (a); amino acids (b); and lipids (c). Based on genes identified in genome of *Thraustochytrium* sp. RT2316-16. Results were obtained using KEGG Mapper Reconstruction tool.

Figure S6. Terpenoid backbone biosynthesis in *Thraustochytrium* sp. RT2316-16. Results were obtained with the KEGG Mapper Reconstruction tool. Red boxes denote enzyme coded by the genes annotated in the genome.

Figure S7. Pairwise sequence alignment between Thraus_T3283 and *crtIBY Aurantiochytrium* sp. KH105 (accession BBB35234.1) genes. Conserved domains are highlighted. Conserved Domains Database (CDD) tool at NCBI [60] was used to identify conserved domains.

Table S1. Some morphological characteristics of the isolated thraustochytrid strains. Colony texture: soft (S) or firm (F); colony size: punctiform (P) or small (S); color of the colony: white (W), light yellow (LY), or orange (O); growth in liquid medium (LM) as: individual (I) or grouped (G) cells; presence of motile zoospores (MZ).

**Strain**	**Closest relative in GenBank**	**Colony texture**	**Colony size**	**Color**	**Growth in LM**	**MZ**
RT2316-14	*Oblongichytrium* sp.	S	P	W	I	Yes
RT2316-15	*Oblongichytrium* sp.	F	S	W	I	No
RT2316-18	*Thraustochytrium* sp.	S	P	W	I	Yes
RT2316-21	*Oblongichytrium* sp.	F	S	W	I	No
RT2316-22	*Oblongichytrium* sp.	F	S	W	I	No
RT2316-23	*Oblongichytrium* sp.	F	S	W	I	Yes
RT2316-24	*Oblongichytrium* sp.	F	S	W	I	No
RT2316-25	*Oblongichytrium* sp.	F	S	W	I	No
RT2316-26	*Oblongichytrium* sp.	F	S	W	I	Yes
RT2316-28	*Aurantiochytrium* sp.	S	P	W	I	No
RT2316-29	*Oblongichytrium* sp.	S	S	W	I	No
RT2316-31	*Oblongichytrium* sp.	S	S	W	I	No
RT2316-37	*Thraustochytrium* sp.	F	S	O	G	No
RT2316-38	*Thraustochytrium* sp.	S	S	LY	I	No
RT2316-16	*Thraustochytrium* sp.	S	S	O	G	No
RT2316-45	*Thraustochytrium* sp.	F	S	LY	G	No
RT2316-44	*Thraustochytrium* sp.	F	S	LY	G	No
RT2316-17	*Thraustochytrium* sp.	F	S	O	I	No
RT2316-42	*Thraustochytrium* sp.	F	S	O	G	No
RT2316-40	*Thraustochytrium* sp.	S	P	LY	I	No
RT2316-49	*Thraustochytriidae* sp.	F	S	LY	G	No
RT2316-50	*Aurantiochytrium* sp.	S	S	LY	I	No

Table S2. Effects of initial glucose concentration (G_0) on biomass concentration (X) and the content of total lipids (TL) and carotenoids (TC) in biomass of *Thraustochytrium* sp. RT2316-16 after 5 days of culture.

G_0 (g/L)	X (g/L)	TL (%)$^{£}$	TC (μg/g)$^{£}$	GC (%)$^{¥}$
20	9.7±1.8	36.7±2.3^{b}	60.8±0.3^{a}	94.6
30	9.1±0.5	46.8±2.1^{a}	51.7±3.4^{b}	79.3
40	10.1±0.6	36.9±2.0^{b}	51.8±4.9^{b}	63.5
50	9.9±1.9	34.1±2.1^{b}	51.6±1.3^{b}	51.0

$^{£}$ A different superscript letter within a column denotes significant differences ($p < 0.05$).
$^{¥}$ Glucose consumption (GC) is the percentage of the initial glucose (G_0) consumed by the biomass.

Table S3. *Thraustochytrium* sp. RT2316-16 genes annotated for biosynthesis, elongation and desaturation of fatty acids.

Enzyme (reaction)	EC number	Swiss Prot ID
**Fatty acid biosynthesis**		
Fatty acid synthase subunit β	2.3.1.86	FAS1_YARLI; ORYB_ASPOR
Fatty acid synthase subunit α	2.3.1.86	FAS2_YEAST
Malonyl CoA-acyl carrier protein transacylase	2.3.1.39	FABD_BACSU; FABD_HUMAN
Acetyl-CoA carboxylase	6.4.1.2	ACAC_DICDI; ACACA_BOVIN; ACACA_RAT
3-Oxoacyl-[acyl-carrier-protein] synthase	2.3.1.179	KASM_ARATH
3-Oxoacyl-[acyl-carrier-protein] reductase FabG	1.1.1.100	FABG_THEMA
Enoyl-[acyl-carrier-protein] reductase [NADH] FabI	1.3.1.9; 1.3.1.10	FABI_SYNY3
Long-chain-fatty-acid--CoA ligase	6.2.1.3	LCFB_BACSU; ACSL3_PONAB
Long-chain acyl-CoA synthetase	6.2.1.3	LACS7_ARATH
**Biosynthesis of unsaturated fatty acids**		
Elongation of very long chain fatty acids protein 2 (ELOV2)[¥]	2.3.1.199	ELOH2_SCHPO
Elongation of very long chain fatty acids protein 4 (ELOV4)[£]	2.3.1.199	ELOV4_MOUSE

Elongation of very long chain fatty acids protein 5 (ELOV5)[£]	2.3.1.199	ELOV5_XENTR
Elongation of very long chain fatty acids protein 6 (ELOV6)[‡]	2.3.1.199	ELOV6_MOUSE; ELOV6_CHICK
Very-long-chain 3-oxoacyl-CoA reductase	1.1.1.330	DHB12_BOVIN; KCR1_ARATH; KCR2_ARATH
Very-long-chain (3R)-3-hydroxyacyl-CoA dehydratase	4.2.1.134	HACD_CAEEL
Very-long-chain enoyl-CoA reductase	1.3.1.93	None
Acyl-CoA desaturase (Δ9 desaturase)	1.14.19.1	FAT7_CAEEL
Delta(12) fatty acid desaturase FAD2	1.14.19.6	FAD2B_CALOF
Delta(8)-fatty-acid desaturase	1.14.19.3	SLD2_ARATH
Acyl-lipid (8-3)-desaturase	1.14.19.44	D5FAD_THRSP
Acyl-lipid (7-3)-desaturase (Δ4 desaturase)	1.14.19.31	D4FAD_THRSP

[¥] ELOVL2 acts specifically on polyunsaturated acyl-CoA with a higher activity toward C20:4n-6 and EPA-CoAs, among others [34]. Other substrates include DTA-CoA, EPA-CoA, DPA-CoA.

[£] ELOLV4 substrates: DTA-CoA, C26:4n6-CoA, C28:4n6-CoA, C30:4n6-CoA, C32:4n6-CoA, C34:4n6-CoA, C34:6n6-CoA, C24:0-CoA, C26:0-CoA, C28:0-CoA, C30:0-CoA, DHA-CoA, C24:5n3-CoA, C24:6n3-CoA, C26:5n3-CoA, C26:6n3-CoA, C28:5n3-CoA, C28:6n3-CoA, C30:5n3-CoA, C30:6n3-CoA, C32:5n3-CoA, C32:6n3-CoA, C34:5n3-CoA, C34:6n3-CoA, C36:5n3-CoA.

[‡] ELOLV6 substrates: C12:0-CoA, C14:0-CoA, C16:0-CoA, C16:1-CoA, C18:1-CoA, C18:2n-6-CoA, C18:3n-3-CoA.

‡ Gene Thraus_T4048 was translated to protein, and queried by homology against non-redundant protein database in NCBI using BLASTP algorithm (https://blast.ncbi.nlm.nih.gov). The results showed a high identity match (63.9%) with a Δ5-desaturase of *Thraustochytrium aureum* (accession BAK08911.1).

Table S4. *Thraustochytrium* sp. RT2316-16 genes annotated for biosynthesis of terpenoid backbone and carotenoids.

Enzyme (reaction)	EC number	Swiss Prot ID
Acetyl-CoA acetyltransferase	2.3.1.9	THIL_ALLVD; THIC1_ARATH; THIL_XENTR
Hydroxymethylglutaryl-CoA synthase A	2.3.3.10	HMCSA_DICDI
3-Hydroxy-3-methylglutaryl-coenzyme A reductase 2	1.1.1.34	HMDH2_DICDI
Mevalonate kinase	2.7.1.36	MVK_THEKO
Diphosphomevalonate decarboxylase	4.1.1.33	MVD1_MOUSE
Isopentenyl-diphosphate Δ-isomerase 1	5.3.3.2	IDI1_BOVIN
Farnesyl pyrophosphate synthase	2.5.1.1; 2.5.1.10	FPPS_YEAST
Geranylgeranyl pyrophosphate synthase	2.5.1.1; 2.5.1.10; 2.5.1.29	GGPPS_MOUSE
Probable hexaprenyl pyrophosphate synthase, mitochondrial	2.5.1.82; 2.5.1.83	COQ1_NEUCR
Carotenoid 3,4-desaturase[⁎]	1.3.99.37	CRTD_HALJT
Cytochrome P450 3A12	1.14.14.1	CP3AC_CANLF

‡ A blast search in NCBI showed a 59% identity match to β-carotene synthase of *Aurantiochytrium* sp. KH105 (accession BBB35234.1).

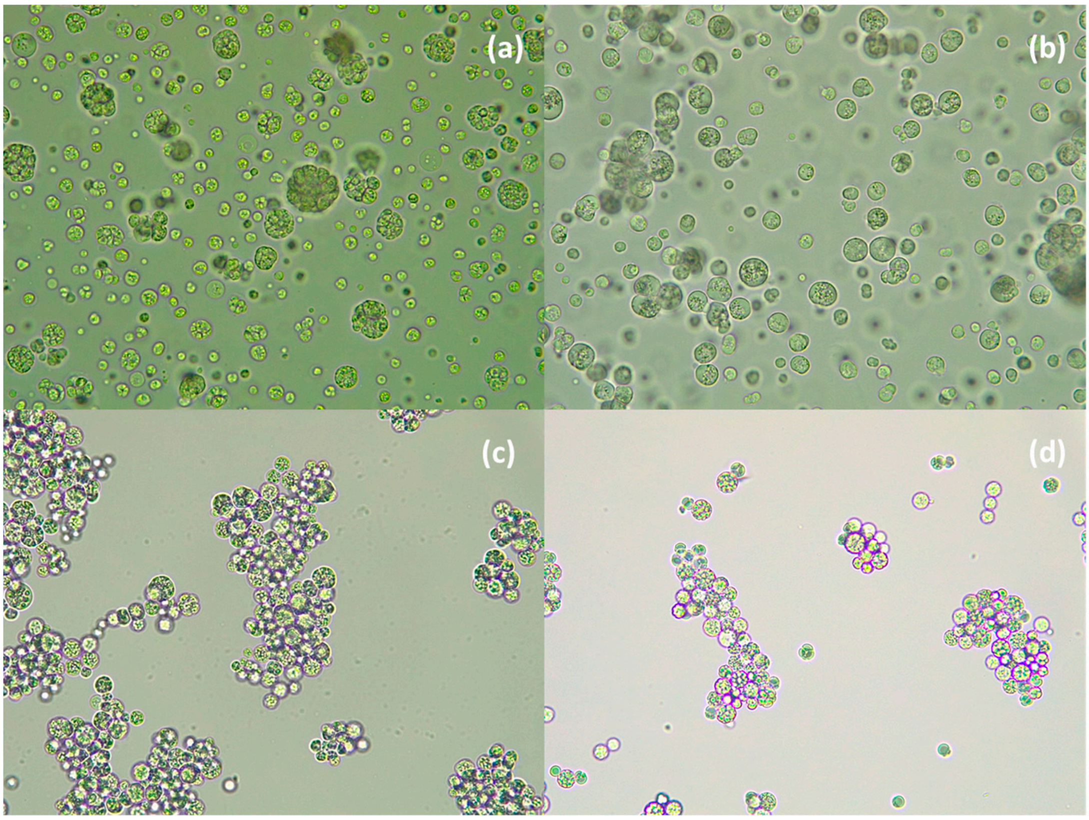

Figure S1. Light microscope image (40×) of some of the isolated thraustochytrid strains. (a) RT2316-29, (b) RT2316-15, (c) RT2316-45 and (d) RT2316-16.

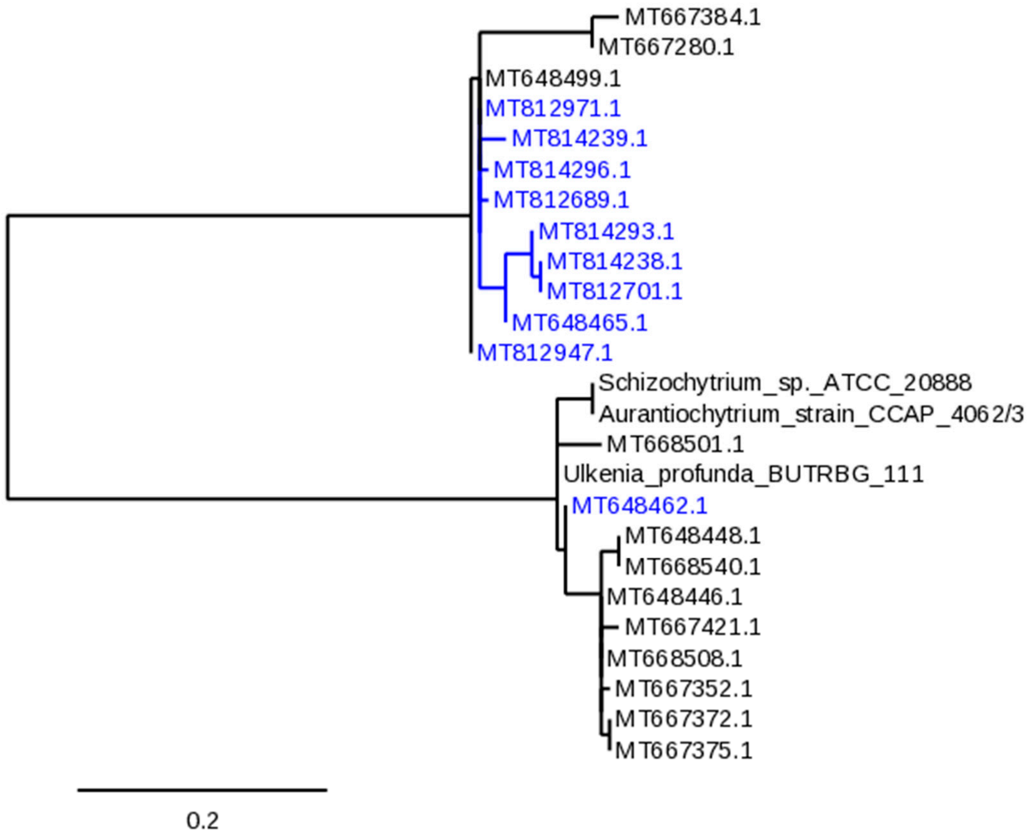

Figure S2. Phylogenetic analysis of Antarctic thraustochytrids isolated from samples collected at different locations in Antarctica during Antarctic Scientific Expedition 54, February 2018 (Tables 1 and 2). Phylogenetic tree was generated by phylogeny.fr [41] (http://www.phylogeny.fr), using MUSCLE, ProtDist/FastDist+BioNJ (distance-based method) and TreeDyn, for multiple sequence alignment, tree construction and tree visualization, respectively. Names shown in blue are isolates that accumulated carotenoids.

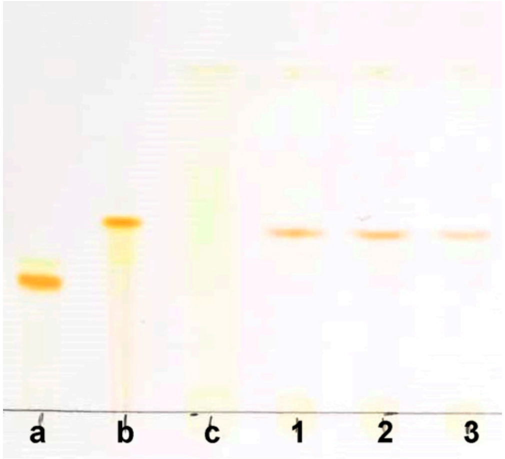

Figure S3. Thin layer chromatogram of carotenoids recovered from *Thraustochytrium* sp. RT2316-16 biomass (lanes 1–3) and authentic standards (astaxanthin, lane a; canthaxanthin, lane b; β-carotene, lane c).

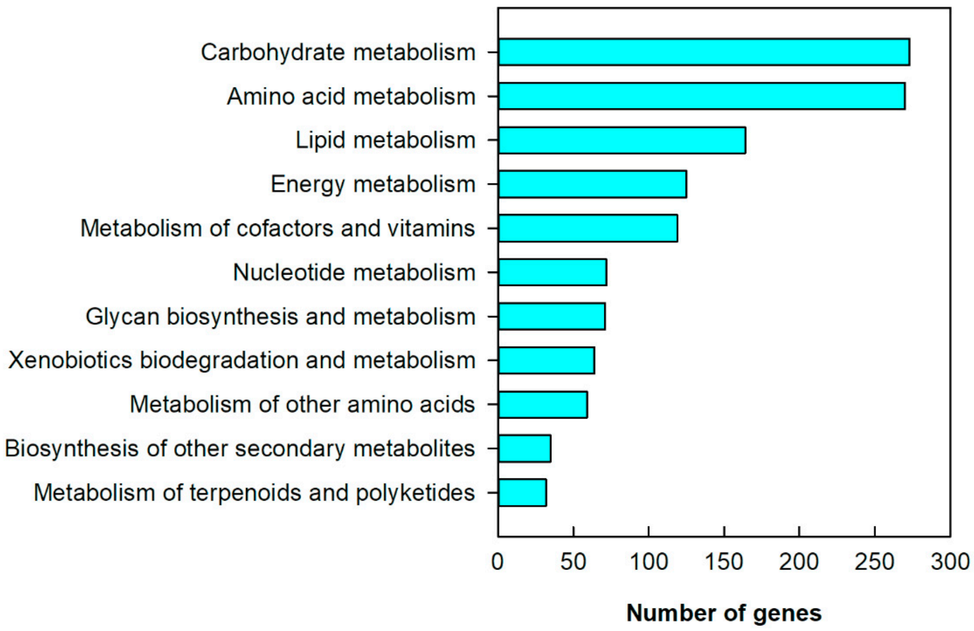

Figure S4. Distribution of enzymes among the metabolic pathways of *Thraustochytrium* sp. RT2316-16, based on the enzyme genes identified in the genome. Results were obtained using KEGG Mapper Reconstruction tool.

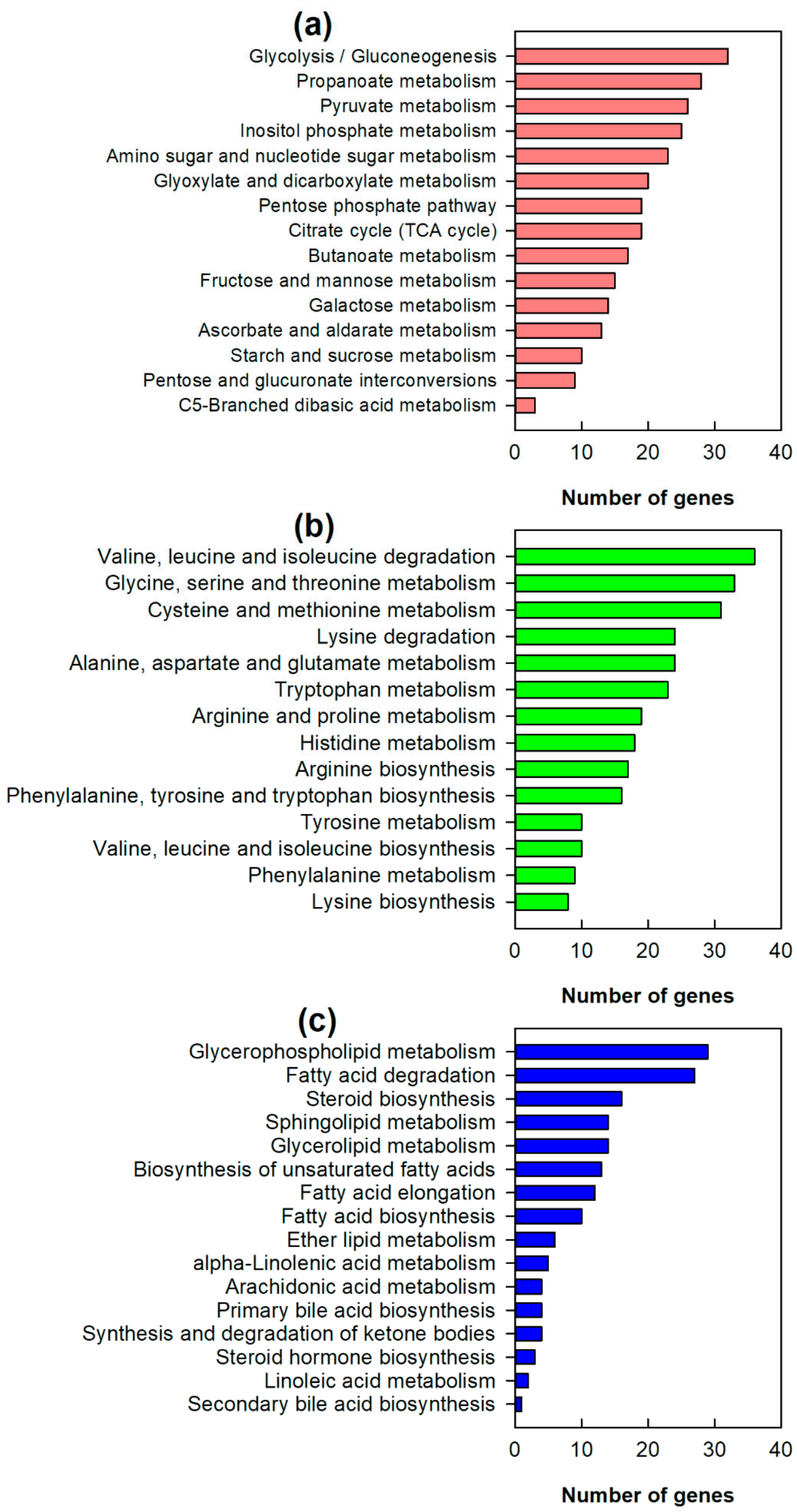

(a)
Glycolysis / Gluconeogenesis
Propanoate metabolism
Pyruvate metabolism
Inositol phosphate metabolism
Amino sugar and nucleotide sugar metabolism
Glyoxylate and dicarboxylate metabolism
Pentose phosphate pathway
Citrate cycle (TCA cycle)
Butanoate metabolism
Fructose and mannose metabolism
Galactose metabolism
Ascorbate and aldarate metabolism
Starch and sucrose metabolism
Pentose and glucuronate interconversions
C5-Branched dibasic acid metabolism
0
10
20
30
40
Number of genes
(b)
Valine, leucine and isoleucine degradation
Glycine, serine and threonine metabolism
Cysteine and methionine metabolism
Lysine degradation
Alanine, aspartate and glutamate metabolism
Tryptophan metabolism
Arginine and proline metabolism
Histidine metabolism
Arginine biosynthesis
Phenylalanine, tyrosine and tryptophan biosynthesis
Tyrosine metabolism
Valine, leucine and isoleucine biosynthesis
Phenylalanine metabolism
Lysine biosynthesis
0
10
20
30
40
Number of genes
(c)
Glycerophospholipid metabolism
Fatty acid degradation
Steroid biosynthesis
Sphingolipid metabolism
Glycerolipid metabolism
Biosynthesis of unsaturated fatty acids
Fatty acid elongation
Fatty acid biosynthesis
Ether lipid metabolism
alpha-Linolenic acid metabolism
Arachidonic acid metabolism
Primary bile acid biosynthesis
Synthesis and degradation of ketone bodies
Steroid hormone biosynthesis
Linoleic acid metabolism
Secondary bile acid biosynthesis
0
10
20
30
40
Number of genes

Figure S5. Distribution of enzymes within the following metabolisms: carbohydrates (a); amino acids (b); and lipids (c). Based on genes identified in genome of *Thraustochytrium* sp. RT2316-16. Results were obtained using KEGG Mapper Reconstruction tool.

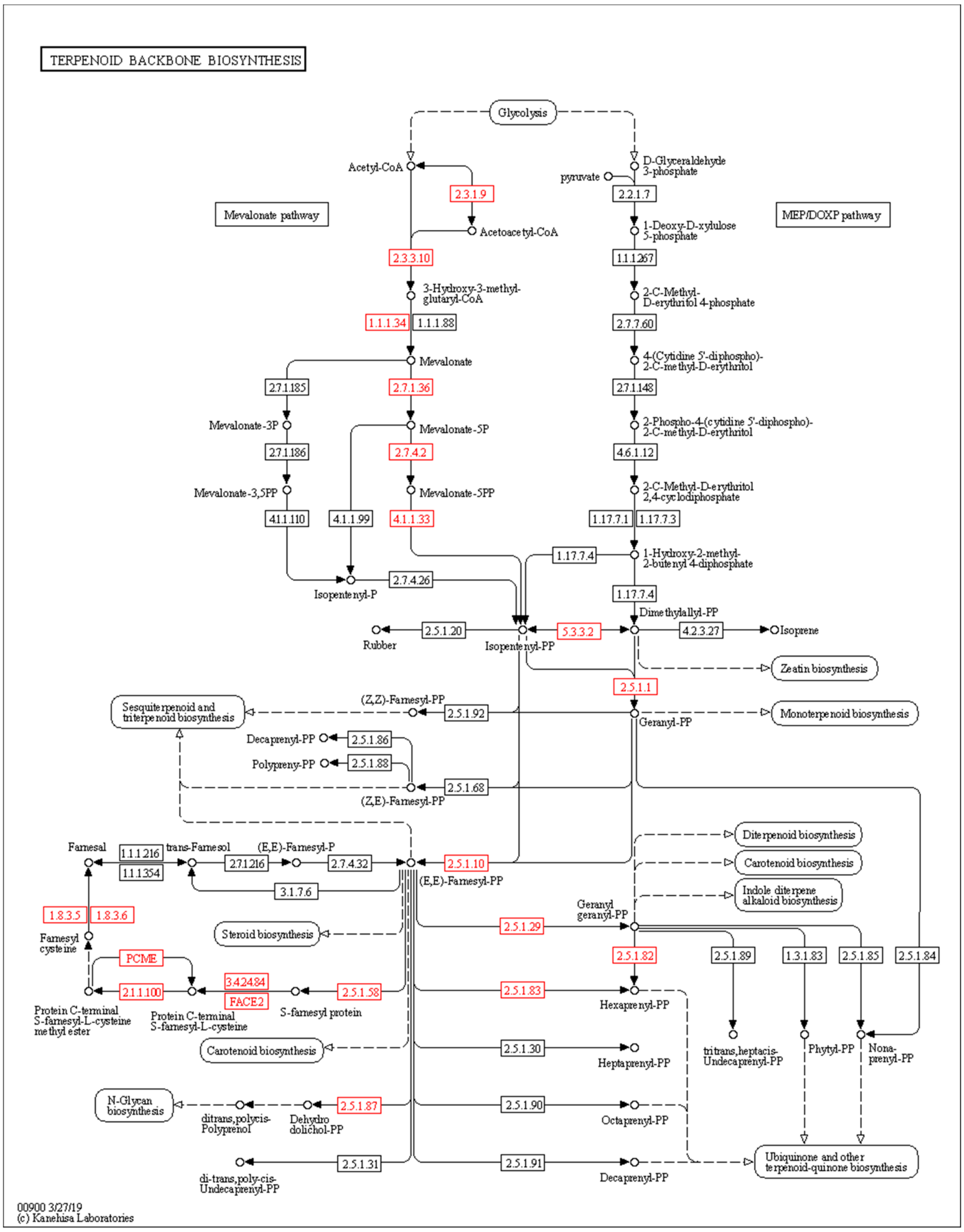

Figure S6. Terpenoid backbone biosynthesis in *Thraustochytrium* sp. RT2316-16. Results were obtained with the KEGG Mapper Reconstruction tool. Red boxes denote enzymes coded by the genes annotated in the genome.

```
                                                        ctrl superfamily | Bacterial-type phytoene desaturase
Thraustochytrium sp. RT2316-16   49  QHIAVLGAGYAGLAAACELRRLGYAVTVYERNAFVGGRAHQFEAAG-----FTFDAGPSW  103
                                     + IAVLGAGYAGL+AACEL RLG+ V V E+N +VGGRAHQFE        F FDAGPSW
    Aurantiochytrium sp. KH105   51  KRIAVLGAGYAGLSAACELSRLGHEVVVLEKNDYVGGRAHQFEVEADNGQTFKFDAGPSW  110
Thraustochytrium sp. RT2316-16  104  YWMPEVFDRFFARFGRRTREFYSITRLDPAYRVIGRSRHGAAAIDVPGTRA-GYMAWARR  162
                                     YWMPEVFDRFFAR+GR  +EFY + RLDPAYR+I     +G    +DVPG  +   +M+WAR+
    Aurantiochytrium sp. KH105  111  YWMPEVFDRFFARYGRTVQEFYQLERLDPAYRIIRNDHNGEGTVDVPGASSEAFMSWARQ  170
Thraustochytrium sp. RT2316-16  223  RRFVSDPTLLMTLKWPVIFLGASPKEAPALYSLMTYAGHALGTWYPSGGMTSPAKAMAAM  282
                                     ++++S  TLLM LKWPVIFLGASP  APALYS+MTY GHALGT+YP+GG+   P   A+A +
    Aurantiochytrium sp. KH105  230  QKYISSDTLLMILKWPVIFLGASPNGAPALYSMMTYGGHALGTFYPTGGLARPVVAIAEL  289
Thraustochytrium sp. RT2316-16  283  ARDMGVQIRLSAEVTSIKFDKTGEGSRASHVLGAAFQDPVDGIVGAGDYYHTEQKLLPPR  342
                                     A+D+GV I+L AEVTS +FD+TG G +A          +  VDG+V A DY+H EQ LLPP
    Aurantiochytrium sp. KH105  290  AKDLGVDIQLDAEVTSFRFDETGRGVQAVCTRNDRCE-AVDGVVAAADYHHVEQTLLPPE  348
Thraustochytrium sp. RT2316-16  343  LRRYDARYWERQVLSPSCLLFYLGVNRRVEGLLHHTFFFDEDLDAHLAAAFERHEHSDRP  402
                                     LRRY+   +W+ QV+SPSC+LFYLG + R++GL HHTFFFD DLDAHL AAF+ H  ++ P
    Aurantiochytrium sp. KH105  349  LRRYEQGFWDAQVMSPSCVLFYLGFDHRIQGLTHHTFFFDRDLDAHLHAAFDTHTWAEEP  408
Thraustochytrium sp. RT2316-16  402  TFYVSATSKTDPSTRPDGDQGEALFVLVPISYRLNGTDTEALRRAVLHKVLERMERALGE  462
                                      FYVSATSKTDPS      QGEALFVLVPISY+LNGTD  A R  +LH VL RME  L +
    Aurantiochytrium sp. KH105  406  VFYVSATSKTDPSVVTG--QGEALFVLVPISYQLNGTDNAARREQILHTVLTRMEENLKQ  466
Thraustochytrium sp. RT2316-16  463  PIRSALTYTRMYGPSDFAEEFHSFRGNAFGHANILSQSLILKPSMDSLADNIVFAGHLTN  522
                                     P+R  L Y + YG +DF  +FHSFRGNAFGHAN LSQSL+LKPSMDSL +N+VFAGHLTN
    Aurantiochytrium sp. KH105  467  PLREWLVYQKSYGTTDFERDFHSFRGNAFGHANTLSQSLVLKPSMDSLLNNLVFAGHLTN  526
Thraustochytrium sp. RT2316-16  523  PGPGVPPSIVSGTVAAGLLDVKLLDCERIEDPASHYLLTAAQQHRRARLCAARPASQGR  582
                                     PGPGVPPSIVSGTV+A LL        ++++  A+H+LL
    Aurantiochytrium sp. KH105  527  PGPGVPPSIVSGTVSANLLH------DKLQVTANHHLLG--------------------
                                                                       Squalene / Phytoene synthase
Thraustochytrium sp. RT2316-16  583  ELFKWGLAALAGLHVLAFAWVMVSARRRSYLLAVKLLFEHGRTYFAAATLMNLGAFLDTA  642
                                      F    A L  L +        + S R  SY+  ++LL+ HGRTYFAAATLM   AFLDTA
    Aurantiochytrium sp. KH105  560  --FTLLGALLGALFLGILTLSVFSTRFVSYVECIRLLYVHGRTYFAAATLMKPMAFLDTA  617
Thraustochytrium sp. RT2316-16  643  AMYALFRVADDFVDNEDAAAQRHANLETFIADFWRCWESGTGDYSLHPTLPAIVESARRH  702
                                     AMY LFRVADD+VDN    A +R  NL+ F+ADFWRCWESG GDY+ HPTLPAI+ESA R
    Aurantiochytrium sp. KH105  618  AMYGLFRVADDYVDNVGDAGERQRNLDAFMADFWRCWESGRGDYARHPTLPAIIESAHRR  677
Thraustochytrium sp. RT2316-16  703  RYPRDLFERFFRSMRMDVG-DLVCETLDDTMDYMEGSAAVIGEFMLPVLMPAAAKSQVDR  761
                                      YPR+LFERFFRSMRMD     +VC  T+DDTM+YMEGSAAVIGEFMLP+LMP           +
    Aurantiochytrium sp. KH105  678  AYPRELFERFFRSMRMDAKRKVVCLTMDDTMEYMEGSAAVIGEFMLPILMPDRDSLAFKQ  737
Thraustochytrium sp. RT2316-16  762  AMPHARDLGLAFQLTNMIR  780
                                     A+PHARDLGLAFQ+TNM+R
    Aurantiochytrium sp. KH105  738  AIPHARDLGLAFQITNMLR  756
```

Figure S7. Pairwise sequence alignment between Thraus_T3283 and *crtIBY* *Aurantiochytrium* sp. KH105 (accession BBB35234.1) genes. Conserved domains are highlighted. Conserved Domains Database (CDD) tool at NCBI [60] was used to identify conserved domains.
